# The relationship of plasma renin, angiotensin, and aldosterone levels to blood pressure variability and target organ damage in children with essential hypertension

**DOI:** 10.1186/s12872-020-01579-x

**Published:** 2020-06-16

**Authors:** Yang Liu, Yao Lin, Ming-Ming Zhang, Xiao-Hui Li, Yan-Yan Liu, Jing Zhao, Lin Shi

**Affiliations:** 1 Capital Institute of Pediatrics-Peking University Teaching Hospital, Beijing, 100191 China; 2 Department of Cardiology, Children’s Hospital Affiliated to Capital Institute of Pediatrics, Beijing, 100020 China

**Keywords:** Blood pressure monitoring, Child, Hypertension, Renin-angiotensin-aldosterone system

## Abstract

**Background:**

To investigate the relationships of plasma renin, angiotensin, and aldosterone levels to blood pressure variability and target organ damage in children with essential hypertension.

**Methods:**

A case-control study was conducted on 132 children diagnosed with essential hypertension (103 males and 29 females with the mean age of 11.8 ± 2.4 years). The plasma RAAS levels were measured using the enhanced chemiluminescence method, the ambulatory blood pressure was monitored for 24 h, and then the average real variability (ARV) was calculated. Data on indicators were used for assessing cardiac and renal damages. The correlations of plasma renin, angiotensin, and aldosterone (RAAS) levels to blood pressure variability (BPV) and target organ damage (TOD) were studied. A comparison between the groups was conducted using SPSS 20.

**Results:**

Among the 132 children, 55 cases had target organ damage. The 24-h ARV and the daytime ARV of the systolic blood pressure of the high angiotensin II (AT II) group was significantly higher than that of the normal AT II group (t = 2.175, *P =* 0.031; t = 2.672, *P =* 0.009). Plasma AT II and aldosterone levels were significantly associated with the left ventricular mass index (*r* = 0.329, *P =* 0.0001; *r* = 0.175, *P =* 0.045). Linear regression analysis showed that AT II [*β ± s.e. =* 0.025 ± 0.006, *95% CI (*0.013–0.038), *P =* 0.0001] and aldosterone [*β ± s.e. =* 0.021 ± 0.007, *95% CI (*0.008–0.034), *P =* 0.002] were risk factors for LVH.

**Conclusions:**

The AT II level in children with essential hypertension affected the variability of the 24-h and the daytime SBP. Plasma AT II and aldosterone levels were associated with cardiac damage. Results from this study indicated that AT II and aldosterone are risk factors for LVH in childhood hypertension and are of great significance for improving the clinical prognosis of pediatric patients with hypertension.

## Background

Hypertension is a disease that seriously threatens human health by causing damage to multiple target organs, such as the cardiovascular system, kidneys, central nervous system (CNS), and fundus. Many epidemiological studies have focused on identifying factors that increase the risk of hypertension. Essential hypertension is a multi-gene disease in which the genetic environment interacts with humoral factors. The renin-angiotensin-aldosterone system (RAAS) has a pivotal role in regulating blood pressure and water-electrolyte balance. Over-activation of RAAS plays an important role in the pathogenesis of hypertension [1]. An earlier study has found that RAAS genes are correlated with cardiovascular diseases, hypertension, left ventricular hypertrophy (LVH), and myocardial infarction [2]. Ambulatory blood pressure monitoring (ABPM) has been extensively used to monitor 24-h blood pressure changes in hypertensive patients to reveal blood pressure variability (BPV), which refers to the fluctuation of blood pressure over a period of time. It is an objective indicator of blood pressure stability and is affected by many factors. A previous study has shown that BPV in adult hypertensive patients is associated with RAAS. Inhibition of the activation of RAAS helps to lower blood pressure and reduce blood pressure fluctuation, thereby reducing the risk of cardiovascular events [3]. The incidence of essential hypertension in children and adolescents has increased recently. Children and adolescents with high blood pressure are not only well correlated with surrogate outcomes of LVH and early coronary artery disease [4, 5], but also have a greater likelihood of developing adult hypertension, which places these patients at substantial risk for serious cardiovascular disease [6]. Several studies have demonstrated that the components of the RAAS, both angiotensin II and aldosterone, are associated with BPV and involved in the progression of hypertensive target organ damage and cardiovascular diseases in adults [7–9].

However, the relationships of RAAS levels to BPV and target organ damage (TOD) in hypertensive children remain unclear. Hence, we conducted a correlation analysis between RAAS and BPV as well as TOD in children with essential hypertension from August 2017 to June 2019.

## Methods

### Subjects

A total of 132 children, comprising 103 males (78.0%) and 29 females (22.0%) diagnosed with essential hypertension and admitted to the Affiliated Children’s Hospital of Capital Institute of Pediatrics (Beijing, China) from August 2017 to June 2019 were included in this case-control study. The research protocol of this study was approved by the Ethics Committee of the Children’s Hospital Affiliated to Capital Institute of Pediatrics. The parents or guardians of the patients received information about the relevant examinations and signed the written informed consent before the study.

All blood pressure measurements were performed using the auscultation method as recommended by *the Fourth Report on the Diagnosis, Evaluation, and Treatment of High Blood Pressure in Children and Adolescents*, and the results were used for hypertension diagnosis and stage classification [10]. Hypertension was diagnosed when the average systolic and/or diastolic BP was ≥95th percentile from the auscultation measurement on at least 3 separate occasions adjusted for gender, age, and height. Stage 1 hypertension was diagnosed if a child’s BP was greater than the 95th percentile but less than or equal to the 99th percentile plus 5 mmHg; and stage 2 hypertension was diagnosed if a child’s BP was greater than the 99th percentile plus 5 mmHg.

Exclusion criteria for this study were patients older than 18 years old; with secondary hypertension caused by kidney disease, vascular disease, endocrine disease, CNS disease, or drugs; or with essential hypertension that had been treated with antihypertensive drugs.

### Laboratory examinations

#### Blood lipids, electrolytes, and other biochemical indicator measurements

The pediatric patients were fasted for 8 h, followed by the collection of their venous blood in the morning to measure serum sodium and potassium concentrations, plasma cholesterol, triglyceride, uric acid, and other parameters.

#### Plasma renin activity, angiotensin II level, and aldosterone level measurements

Samples of venous blood were taken from the patients in the morning before eating, and the patients were required to have stayed in bed for more than 4 h. The samples were collected in tubes containing anticoagulants and refrigerated. After centrifugation for 5 min (1000 r/min), the plasma was collected. A Maglumi 2000 Plus automatic chemiluminescence immunology analyzer (Snibe Diagnostic) was used to measure angiotensin II, renin, and aldosterone. Renin activity was assayed using a chemiluminescence immune sandwich method, and the levels of angiotensin II and aldosterone were assessed using chemiluminescence immune competition. The normal reference ranges of angiotensin II, renin, and aldosterone were 25–60 pg/mL, 0.15–2.33 ng/mL/hr., and 30–160 pg/mL, respectively.

#### Ambulatory blood pressure measurement

All patients underwent 24 h ambulatory blood pressure monitoring using a DMS-ABP device (DM Software Inc., Beijing). Information on the procedure and the device was provided to the patients. In order for the device to function properly, patients were told to perform their daily activities normally, but to remain immobile during measurements. BP recordings were programmed to occur every 30 min during the day and every 60 min while sleeping.

Sleep and wake times were recorded and adjusted for each patient to define the nighttime period. On the basis of these measurements, 24-h systolic blood pressure (24-h SBP), 24-h diastolic blood pressure (24-h DBP), daytime SBP, daytime DBP, nighttime SBP and nighttime DBP were determined. The method was considered reliable if > 75% of the measurements were valid.

Blood pressure variability was calculated using the average real variability (ARV) index;

ARV was calculated mathematically using the numerical variation between two successive measurements, as shown in the formula below:

$$ \mathrm{ARV}=\frac{1}{N-1}\sum \limits_{k=1}^{N-1}\left|{\mathrm{BP}}_{k+1}-{\mathrm{BP}}_k\right|, $$

where N is the number of valid blood pressure measurements, and BP_k + 1_ and BP_k_ represent two successive blood pressure measurements. The rationale for selecting the ARV index for blood pressure variability calculation was based on earlier reports stating that the ARV index was a more reliable index for establishing the prognostic significance of blood pressure variability [11].

### Indicators for evaluating target organ damage

#### Heart

LVH was assessed using echocardiography. Left ventricular internal dimension (LVIDd), interventricular septal thickness (IVST), and left ventricular posterior wall thickness (LVPWT) at the end diastole were measured using the Philips iE33 Ultrasound System. Left ventricular mass (LVM) was calculated as LVM = 1.04 × 0.8 × ((LVIDd+IVST+LVPWT)^3^–LVIDd^3^) + 0.6 [12]; and LVM index (LVMI) was calculated as LVMI = LVM/height^2.16^; and relative left ventricular wall thickness (RWT) was calculated as RWT = (IVST+LVPWT)/LVIDd; For the diagnosis of LVH, LVMI ≥45 g/m^2.16^ or RWT > 0.41 was considered abnormal [13, 14].

#### Kidneys

Renal damage was diagnosed in the patient if any of the following conditions was fulfilled: albuminuria (urinary albumin/creatinine quotient) > 3 mg/mmol creatinine; 24-h urine protein excretion > 200 mg/m^2^/day [15, 16].

#### Grouping

The patients with essential hypertension were grouped according to their plasma renin activity, or their levels of angiotensin II or aldosterone. Based on the renin activity, patients were classified into a high renin group (> 2.33 ng/mL/hr., *n* = 58) and a normal renin group (≤ 2.33 ng/mL/hr., *n* = 74). Based on the angiotensin II and aldosterone levels, the patients were classified into a high angiotensin II group (> 60 pg/mL, *n* = 87) and a normal angiotensin II group (≤ 60 pg/mL, *n* = 45) as well as a high aldosterone group (> 160 pg/mL, *n* = 70) and normal aldosterone group (≤ 160 pg/mL, *n* = 62). Also, the patients were grouped according to the presence or absence of target organ damage. The patients in the organ damage-group were subdivided into cardiac-damage and renal-damage groups. The differences in plasma renin, angiotensin, and aldosterone levels between groups were analyzed.

### Statistical analysis

SPSS 20.0 software (IBM, Armonk, NY) was used for data processing in this study. Normally distributed measurement data are shown as mean ± standard deviation. Independent sample *t-*test was used to compare the difference between two groups. Analysis of variance (ANOVA) was used to compare the measured differences among multiple groups. Count data are presented as percentages (%), and the chi-square (χ^2^) test was used to compare the difference between groups. Univariate associations between the indices of RAAS levels, LVMI, and other variables of TOD were assessed through simple linear regression analyses and Pearson correlation coefficients. *P <* 0.05 was considered statistically significant.

## Results

### Demographics, laboratory characteristics and ambulatory blood pressure measurements of the study population

Among the 132 children with hypertension enrolled in this study, there were 103 males (78.0%) and 29 females (22.0%), with a mean age of 11.9 ± 2.1 years (11.7 ± 2.1 years for males and 12.7 ± 2.2 years for females), average height of 164.5 ± 12.5 cm, average body weight of 74.3 ± 22.4 kg, average body mass index (BMI) of 26.9 ± 5.7 kg/m^2^. Among them, 98 patients (74.2%) had their BMI over the 95th percentile of children of the same age and gender and were diagnosed as obese. There were 67 patients (50.8%) with stage 1 hypertension and 65 patients (49.2%) with stage 2 hypertension. The average 24-h SBP and average 24-h DBP of the 132 patients were 125.1 ± 9.7 mmHg and 70.7 ± 6.5 mmHg, respectively. Among them, 55 patients (41.7%) had no clinical symptoms of hypertension, but high blood pressure was found during physical examinations; while 77 patients (58.3%) had clinical symptoms of hypertension, such as dizziness, headaches, nausea, vomiting, chest tightness, or fatigue. All 132 patients with essential hypertension were treated in our hospital for the first time and had no previous drug intervention given.

Demographic, laboratory characteristics and ambulatory blood pressure measurements are shown in Table 1. The comparison of clinical and laboratory characteristics, including age, gender, BMI, blood lipids, serum uric acid, serum sodium concentration, and serum potassium concentration, showed that there were no significant differences between the high and normal renin groups, between the high and normal angiotensin II groups, or between the high and normal aldosterone groups. Comparisons of ambulatory blood pressure measurements showed that there was no difference in the 24-h SBP and 24-h DBP. Comparisons of the 24-h, daytime, and nighttime ARV between the high angiotensin II and normal angiotensin II groups showed that the 24-h and daytime SBP- ARV of the high angiotensin II group was significantly higher than that of the normal angiotensin II group (t = 2.175, *P =* 0.031; t = 2.672, *P =* 0.009). No significant differences in the 24-h, daytime, and nighttime ARV were found between the high renin and normal renin group or between the high aldosterone and normal aldosterone groups.

**Table 1:** Comparison of demographic, laboratory characteristics, and ambulatory blood pressure measurements in different groups of plasma renin activity, angiotensin II, or aldosterone levels

	High renin group (*n* = 58)	Normal renin group (*n* = 74)	High angiotensin II group (*n* = 87)	Normal angiotensin II group (*n* = 45)	High aldosterone group (*n* = 70)	Normal aldosterone group (*n* = 62)
Age (year)	11.46 ± 2.49	12.27 ± 1.78	11.93 ± 2.14	11.88 ± 2.21	11.46 ± 2.46	12.27 ± 2.31
Gender (male)	48 (82.8%)	55 (74.3%)	70 (80.5%)	33 (73.3%)	56 (80%)	47 (75.8%)
BMI (Kg/m^2^)	25.84 ± 4.83	27.81 ± 6.19	26.94 ± 5.50	26.96 ± 6.13	26.00 ± 4.99	27.82 ± 6.22
CHOL (mmol/L)	3.95 ± 0.74	3.93 ± 0.53	3.93 ± 0.63	3.95 ± 0.63	3.98 ± 0.70	3.89 ± 0.52
TG (mmol/L)	1.33 ± 0.82	1.32 ± 0.72	1.26 ± 0.60	1.46 ± 1.04	1.26 ± 0.60	1.41 ± 0.91
UA (μmol/L)	388.60 ± 91.74	416.46 ± 91.82	405.19 ± 91.65	402.52 ± 95.16	386.07 ± 89.58	426.37 ± 91.84
Blood sodium (mmol/L)	141.67 ± 1.99	141.77 ± 1.45	141.99 ± 1.70	141.21 ± 1.60	141.66 ± 1.97	141.80 ± 1.31
Blood potassium (mmol/L)	4.20 ± 0.30	4.23 ± 0.29	4.20 ± 0.31	4.25 ± 0.25	4.26 ± 0.31	4.16 ± 0.28
24-h SBP (mmHg)	124.96 ± 10.70	125.20 ± 8.76	124.95 ± 9.28	125.37 ± 10.36	124.70 ± 9.61	125.57 ± 9.71
24-h DBP (mmHg)	70.84 ± 7.85	70.62 ± 5.41	70.64 ± 6.20	70.86 ± 7.33	70.80 ± 7.38	70.62 ± 5.52
24-h SBP-ARV (mmHg)	10.21 ± 2.82	9.21 ± 2.74	10.29 ± 3.01*	9.18 ± 2.27	9.82 ± 2.70	10.02 ± 2.97
24-h DBP-ARV (mmHg)	8.59 ± 1.96	7.91 ± 1.63	8.34 ± 1.94	8.48 ± 1.82	8.18 ± 1.76	8.63 ± 2.02
Daytime SBP-ARV (mmHg)	10.11 ± 3.38	9.05 ± 2.95	10.33 ± 3.76**	8.76 ± 1.67	9.78 ± 3.58	9.80 ± 2.94
Daytime DBP-ARV (mmHg)	8.57 ± 2.50	7.85 ± 2.02	8.31 ± 2.50	8.47 ± 2.17	8.22 ± 2.53	8.52 ± 2.22
Nighttime SBP-ARV (mmHg)	10.40 ± 3.81	9.67 ± 3.66	10.27 ± 3.45	10.02 ± 4.34	9.88 ± 3.26	10.52 ± 4.26
Nighttime DBP-ARV (mmHg)	8.69 ± 3.04	7.83 ± 2.29	8.41 ± 2.79	8.47 ± 3.01	8.12 ± 2.67	8.79 ± 3.04

*BMI* Body mass index, *CHOL* Cholesterol, *TG* Triglyceride, *UA* Uric acid, *SBP* Systolic blood pressure, *DBP* Diastolic blood pressure,

*ARV* Average real variability. *P = 0.031, **P = 0.009

### Evaluation of target organ damages

Evaluations of heart and kidney damage were performed in the 132 children with hypertension. Among the 132 cases, 55 patients (41.7%) had target organ damage, including 47 patients with a single organ damaged and 8 patients with two organs damaged.

#### Heart

Thirty-four (25.8%) of the 132 patients had LVH. Of these patients, 28 had increased LVMI and normal RWT, manifesting as eccentric remodeling; 4 had normal LVMI and increased RWT, with concentric remodeling; and 2 cases had increased LVMI and RWT, manifesting as concentric hypertrophy.

#### Kidneys

Twenty-nine (22.0%) of the 132 patients had renal damage, one child (0.8%) had elevated 24-h urine protein, and 29 (22.0%) had Albuminuria.

### Relationship between plasma renin-angiotensin-aldosterone levels and target organ damage

Comparison of baseline characteristics of the target organ damage group and the group with no target organ damage. Of the 132 patients, 55 (41.7%) had target organ damage, including 42 males (76.4%) and 13 females (23.6%), with a mean age of 12.4 ± 2.4 years; 77 patients (58.3%) had no target organ damage, including 61 males (79.2%) and 16 females (20.8%), with a mean age of 11.5 ± 2.4 years. No significant differences in gender or age were found between the two groups of patients (χ^2^ = 0.696, *P =* 0.831; t = 1.275, *P =* 0.205).

Correlation analysis between plasma renin/angiotensin/aldosterone levels and target organ damage in 132 pediatric patients. As shown in Table 2, Plasma angiotensin II and aldosterone levels were significantly correlated with LVMI (*P <* 0.05), but not with kidney damage (*P* > 0.05). Further analysis using the Pearson’s correlation coefficient showed that the plasma angiotensin II level was positively correlated with LVMI (r = 0. 329, *P =* 0.0001). Linear regression analysis showed that, adjusted by gender, age, and BMI, AT II [*β ± s.e. =* 0.025 ± 0.006, *95% CI (*0.013–0.038), *P =* 0.0001] and aldosterone [*β ± s.e. =* 0.021 ± 0.007, *95% CI (*0.008–0.034), *P =* 0.002] were risk factors for LVH (Table 3). There were no significant correlations in RWT and albuminuria between the different groups of plasma renin activity, angiotensin II, or aldosterone levels (*β ± s.e.* < 0.001).

**Table 2:** Correlation analysis between plasma renin-angiotensin-aldosterone levels and target organ damages

Target organ damage	Renin-angiotensin-aldosterone	*r-*value	*P* value
LVMI	Renin	0.036	0.685
	Angiotensin II	0.329	0.0001*
	Aldosterone	0.175	0.045*
RWT	Renin	0.153	0.097
	Angiotensin II	0.014	0.883
	Aldosterone	0.08	0.388
Albuminuria	Renin	0.036	0.684
	Angiotensin II	0.063	0.470
	Aldosterone	0.060	0.493

*LVMI* Ventricular mass index, *RWT* Relative wall thickness

*P<0.05 is considered for statistical analysis

**Table 3:** Linear regression analysis between plasma renin-angiotensin-aldosterone levels and LVMI

Characteristic	*β ± s.e.*	*95% CI*		*P-*value
LVMI	0.016 ± 0.012	−0.008	0.040	0.181
Renin	R^2^ = 0.230			
LVMI	0.025 ± 0.006	0.013	0.038	0.0001*
Angiotensin II	R^2^ = 0.307			
LVMI	0.021 ± 0.007	0.008	0.034	0.002*
Aldosterone	R^2^ = 0.307			

Adjusted by gender, age, and BMI

*LVMI* Ventricular mass index, *95% CI* 95% confidence interval

*P < 0.05 is considered for statistical analysis

## Discussion

Essential hypertension is a multi-gene disease in which the genetic components interact with physiological factors. RAAS plays an important role in the pathogenesis of hypertension. Classical RAAS refers to the production and function of angiotensin and aldosterone. Angiotensinogen is secreted by the liver into the blood circulation. A decapeptide, angiotensin I, is generated by the action of renin produced by renal mesangial cells. Angiotensin I is then further converted into an octapeptide, angiotensin II, by the action of angiotensin-converting enzyme (ACE) in the pulmonary circulation. Angiotensin II promotes vasoconstriction. Aldosterone is secreted by the adrenal cortex and is essential for sodium retention and potassium excretion to promote water and sodium reabsorption, thereby causing water and sodium retention and elevated blood pressure [17]. RAAS plays an important role in regulating vascular tone, water-electrolyte balance, and cardiac and vascular remodeling. Activation of RAAS causes vasoconstriction, stimulates the growth of vascular smooth muscle cells, and increases coronary resistance, leading to elevated ventricular pressure load and wall tension, water-sodium retention, increased sympathetic activity, and significantly elevated hypertension [18]. When the regulatory mechanism of the RAAS is disturbed, this leads to changes in the structure of the wall of blood vessels and increases the extent of target organ damage.

Under normal physiological conditions, blood pressure fluctuations are not too extreme (too high or too low). Under pathological conditions, BPV is affected by increased blood volume and neurological and humoral factors, which leads to greater fluctuation in the blood pressure. Among humoral factors, the plasma renin, angiotensin, and aldosterone levels have important effects on BPV. Baltatu et al. [19] studied the regulation of blood pressure and heart rate variability by RAAS in the brain and showed that RAAS in the brain played an important role in regulating the effect of angiotensin II on the diurnal variation of blood pressure. In this study, we analyzed the correlation of different levels of renin, angiotensin II, and aldosterone to BPV indicators. The results showed that children with high angiotensin II levels had significantly elevated 24-h and daytime SBP- ARV, and the angiotensin II level was correlated with 24-h and daytime SBP-ARV. Thus, children with essential hypertension with high angiotensin II levels showed large fluctuations in SBP.

Myocardial remodeling in hypertension refers to cardiac hypertrophy and fibrosis. Sustained blood pressure load first and mainly affects the left heart, resulting in LVH. However, the increase in LVM is not always related to blood pressure values [20]. Myocardial fibrosis is affected by many factors, but the main influencing factor is the activation of RAAS; while angiotensin II, aldosterone, and hemodynamics are the direct factors in cardiac hypertrophy [21]. The suppression of RAAS independently contributed to the magnitude of LVH regression after eliminating the interference of blood pressure [22, 23]. This study analyzed the correlations between plasma renin/angiotensin/aldosterone levels and LVMI and showed that the levels of angiotensin II and aldosterone were associated with LVMI. The correlations of LVMI to angiotensin and aldosterone were further evaluated using a linear regression analysis. The results showed that the plasma angiotensin II and aldosterone levels in patients with LVH were higher than that in patients without LVH. Angiotensin II and aldosterone were positively correlated with LVMI and were risk factors for LVH in hypertensive patients.

The progression of kidney disease is closely related to the regulation of local RAAS. The action of angiotensin II results in increased glomerular capillary pressure in hypertension with renal damage, resulting in increased glomerular permeability and excessive proteins being filtered through the glomeruli; cells in the proximal convoluted tubule absorb the excessive proteins by pinocytosis, which causes tubular damage, interstitial inflammation and fibrosis, as well as the loss of kidney unit function [24]. However, a full understanding of the underlying pathological process is still lacking. This study analyzed the correlations between plasma renin/angiotensin/aldosterone levels and renal damage in 132 children with hypertension and showed no correlation between them. We speculate that this may be due to the relatively small sample size of this study. In addition, the duration of blood pressure elevation in the children with essential hypertension was short, and severe renal lesions had not yet occurred. Therefore, further clinical studies will be necessary to confirm our hypothesis.

Considering that this was a small-scale study from a single center, the generalizability of our data might be limited. The small patient population and low number of females might also limit the statistical power. In addition, CIMT and PWV are also considered markers of TOD but were not assessed in the study; this is also a limitation of the study. We think that additional prospective research is needed to more fully discern the effect of RAAS activity on BPV and TOD.

## Conclusions

In conclusion, RAAS plays an important role in the pathogenesis of hypertension. Our results showed that plasma angiotensin II levels affected 24-h and daytime SBP fluctuations and affected the regulation of the systolic BPV. Angiotensin II and aldosterone levels were associated with hypertensive cardiac damage. They were not only the predisposing factors for cardiac damage in hypertensive patients but were also involved in the disease progression of cardiac damage. Elevated angiotensin II activity was closely related to hypertensive cardiac damage. The results from this study indicated that angiotensin II and aldosterone are risk factors for LVH in childhood hypertension and are of great significance for improving the clinical prognosis of pediatric patients with hypertension.

## Data Availability

The data sets used and/or analyzed during the study are available from the corresponding author on reasonable request.

## Abbreviations

ARV: Average real variability
RAAS: Renin, angiotensin, and aldosterone
BPV: Blood pressure variability
ABPM: Ambulatory blood pressure monitoring
SBP: Systolic blood pressure
DBP: Diastolic blood pressure
BMI: Body mass index
CHOL: Cholesterol
TG: Triglyceride
UA: Uric acid
TOD: Target organ damage
LVIDd: Left ventricular internal dimension
IVST: Interventricular septal thickness
LVPWT: Left ventricular posterior wall thickness
LVM: Left ventricular mass
LVMI: Ventricular mass index
RWT: Relative wall thickness
CI 95%: Confidence interval
